# Functional trait responses to grazing are mediated by soil moisture and plant functional group identity

**DOI:** 10.1038/srep18163

**Published:** 2015-12-11

**Authors:** Shuxia Zheng, Wenhuai Li, Zhichun Lan, Haiyan Ren, Kaibo Wang

**Affiliations:** 1 State Key Laboratory of Vegetation and Environmental Change, Institute of Botany, Chinese Academy of Sciences, Beijing 100093, China; 2 State Key Laboratory of Soil Erosion and Dryland Farming on the Loess Plateau, Institute of Soil and Water Conservation, Chinese Academy of Sciences, Yangling 712100 Shaanxi, China

## Abstract

Abundant evidence has shown that grazing alters plant functional traits, community structure and ecosystem functioning of grasslands. Few studies, however, have tested how plant responses to grazing are mediated by resource availability and plant functional group identity. We examined the effects of grazing on functional traits across a broad range of species along a soil moisture gradient in Inner Mongolia grassland. Our results showed that trait syndromes of plant size (individual biomass) and shoot growth (leaf N content and leaf density) distinguished plant species responses to grazing. The effects of grazing on functional traits were mediated by soil moisture and dependent on functional group identity. For most species, grazing decreased plant height but increased leaf N and specific leaf area (SLA) along the moisture gradient. Grazing enhanced the community-weighted attributes (leaf N_CWM_ and SLA_CWM_), which were triggered mainly by the positive trait responses of annuals and biennials and perennial grasses, and increased relative abundance of perennial forbs. Our results suggest that grazing-induced species turnover and increased intraspecific trait variability are two drivers for the observed changes in community weighted attributes. The dominant perennial bunchgrasses exhibited mixed tolerance–resistance strategies to grazing and mixed acquisitive–conservative strategies in resource utilization.

Grazing by domestic herbivores is the most dominant land use practice that affects plant growth, community structure, and ecosystem functioning and services in grasslands worldwide^1^,^2^. Functional trait-based approaches have recently emerged as a a promising way to understand plant ecological strategies, plant-herbivore interactions, and their linkages to ecosystem functioning^3^,^4^,^5^,^6^. Plant functional traits can provide important insights into the mechanisms underpinning plant reponses to grazing. For example, plant height, individual biomass, and stem-leaf biomass ratio are associated with biomass allocation and species’ capacity for light competition^7^,^8^, and also linked to plant resistance strategies to grazing^9^,^10^,^11^. Leaf N content and specific leaf area (SLA) are tightly linked to leaf economic spectrum and potential growth rate^12^,^13^, as well as plant tolerance strategies to grazing^9^,^14^,^15^. Several studies, however, have suggested that plant species and trait responses to grazing are largely dependent on site productivity or resource availability^3^,^14^,^16^,^17^,^18^,^19^. Thus, a better understanding the mechanisms underpinning plant responses to grazing mediated by resource availability and linkages to ecosystem functioning is fundamental to biodiversity conservation and sustainable ecosystem management.

Resource availability (e.g. water and nutrients) in the environment is proposed as the major determinant of both the amount and type of plant defense^20^. Grazing usually selects species with resistance strategies in dry and infertile systems and species with tolerance strategies in humid and fertile systems^20^,^21^. Moreover, plant species have developed two opposite resource use strategies (i.e. conservative vs. acquisitive) to improve their competitive abilities under environmental fluctuations^12^,^22^,^23^. Species with resource conservative strategies usually show low SLA, low leaf N content, and long life-span^13^,^23^,^24^. In contrast, species with resource acquisitive strategies generally have high SLA, high leaf N content, and short life-span^5^,^13^,^22^,^23^. It is becoming increasingly clear that understanding the ecological context and complexity of trait mediated species’ strategies can provide new insights into the mechanisms governing plant responses to grazing^25^,^26^. For example, two recent studies proposes that resistance and tolerance may complement each other, hence favoring the presence of mixed defense strategies in plants^25^,^27^, which is contrary to a long-standing prediction that resistance and tolerance are functionally redundant alternative strategies^28^. We further propose that plant species may not only exhibit mixed resistance–tolerance strategies to grazing, but also take mixed acquisitive–conservative strategies to resource variation. Nevertheless, the mixed strategies of plants to grazing and environmental fluctuations have rarely been explored so far^26^,^27^.

Recent studies have suggested that functional trait responses to grazing are not only determined by resource availability^2^,^5^,^10^,^17^,^18^, but also dependent on plant species or functional group identity^19^,^29^,^30^. Few studies, however, have synthetically examined the effects of both resource availability (e.g. soil moisture) and functional group identity on plant trait responses to grazing^2^. Moreover, most studies focused on species-specific mean trait responses to grazing, while the intraspecific trait variability has been largely overlooked^31^. The variations in community-weighted attributes, i.e. the mean of trait values weighted by relative abundance of each species in the community, may arise from species turnover and intraspecific variability through plastic adjustment of plants^31^,^32^,^33^. Up to now, little research has been conducted to determine how species turnover and intraspecific variability drive community functional responses to grazing, in particular, the effect of grazing on intraspecific trait variability remains still unclear.

The arid and semiarid Inner Mongolia grasslands have been historically subjected to heavy grazing by livestock^34^,^35^, where plant growth and primary productivity are extremely limited by water availability^36^. In this study, we examine the effects of grazing on nine functional traits of 276 plant species across three vegetation types (i.e. meadow, meadow steppe, and typical steppe) along a soil moisture gradient in the Xilin River Basin of Inner Mongolia grassland. Specifically, we address the following questions: first, how do plant functional traits across a broad range of species respond to grazing? Second, how do the responses of key functional traits (i.e. plant height, individual biomass, stem-leaf biomass ratio, SLA, and leaf N content) to grazing is affected by soil moisture and plant functional group identity? Third, how do species turnover and intraspecific trait variability drive the responses of community-weighted attributes (e.g. SLA_CWM_) to grazing? To address these questions, we hypothesize that: (1) the decrement in plant height along the soil moisture gradient may be attributable to the negative responses of perennial forbs and annuals and biennials to grazing, while the increments in leaf N content and SLA may be driven by the positive responses of perennial grasses and annuals and biennials. (2) Species turnover and intensified intraspecific trait variability may be responsible for the increased community-weighted SLA_CWM_ in response to grazing. (3) Given that the predominance of perennial grasses may be attributable to their long-term evolutionary adaptation to dry environment and co-evolution with herbivores^35^, we further hypothesize that the perennial grasses may exhibit mixed resistance–tolerance strategies to grazing and mixed acquisitive–conservative strategies to resource variation. We expect that perennial grasses increase SLA values in response to grazing in the wet and fertile meadow steppe, but they decrease SLA values in the dry and infertile typical steppe.

## Results

### Associations among species traits

**Figure 1:**
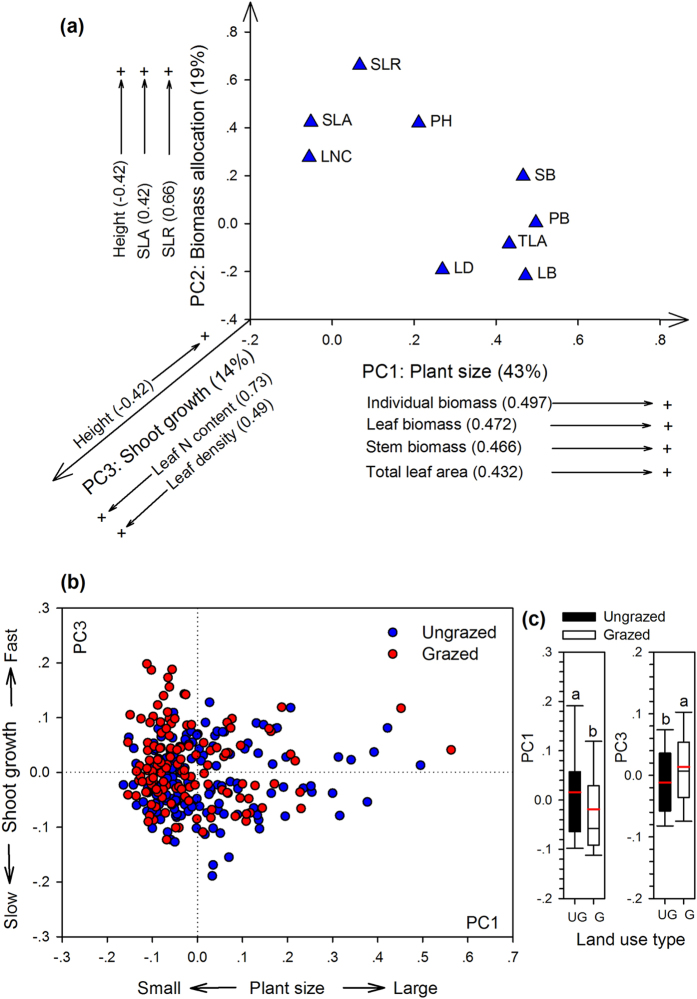
PCA biplot of 276 plant species based on the variance in 9 functional traits explained by the three principal component axes. (**a**) Loading plot of traits. Labels display traits with the highest eigenvector scores on the three principal axes, with the brackets showing the corresponding scores. (**b**) PCA ordination of 149 plant species (blue circles) from the ungrazed and 127 species (red circles) from the grazed communities along PC1 and PC3 axes. (**c**) Box plots illustrate the score distribution of plant species from ungrazed (UG, in black, *n* = 149) and grazed (G, in white, *n* = 127) communities along the PC1 and PC3 axes. Significant differences between the grazed and ungrazed communities along PC1 (*P* = 0.0163) and PC3 (*P* = 0.0016) axes are indicated by different letters. Box plots show the interquartile range, median (black thin line), and mean (red thick line). Abbreviations: PH, plant height; PB, plant individual biomass; SLR, stem-leaf biomass ratio; SB, stem biomass; LB, leaf biomass; TLA, total leaf area; LD, leaf density; SLA, specific leaf area; LNC, leaf N content.

Associations among the 9 functional traits of 276 plant species were analyzed with a PCA (Fig. 1, Supplementary Table S1), and the three principal components together accounted for 76% of the total variance. The first principal component (PC1) explained 43% of the total variance and represented an axis of plant size, which was reflected by plant individual biomass, stem biomass, leaf biomass, and total leaf area. The second principal component (PC2) was strongly associated with plant height, stem-leaf biomass ratio (SLR), and specific leaf area (SLA), accounting for 19% of the total variance. Species found at the positive end of this axis were tall plants with high SLA and more biomass allocation to supportive stem than leaf tissues (high SLR), corresponding to high capacity for aboveground/light competition. The third principal component (PC3), explained an additional 14% of the total variance, which was primarily driven by leaf N content and leaf density, representing an axis of leaf nutrient acquisition and shoot growth (Fig. 1a, Supplementary Table S1). The main trend of trait variation was found between the fast (e.g. high leaf N content and leaf density) and slow shoot growth (e.g. low leaf N content and leaf density).

The PC1 and PC3 axes separated species from the grazed and ungrazed communities (Fig. 1b). Grazing significantly decreased the loading score of plant size along PC1 axis (*P* = 0.0163), but greatly increased that of leaf N content and leaf density along PC3 axis (*P* = 0.0016, Fig. 1c). However, the PC2 score of plant height, SLR and SLA was less affected by grazing (*P* = 0.1011).

### Effects of grazing and soil moisture on plant functional traits

Based on the loading scores along three principal axes, five key functional traits, including plant height, individual biomass, stem-leaf biomass ratio (SLR), SLA, and leaf N content were further selected to examine their responses to grazing along a soil moisture gradient. The generalized linear mixed model analysis showed that plant height, individual biomass and SLA were significantly affected by grazing (Table 1). Four functional traits (except plant height) were greatly influenced by soil moisture. Plant height, SLA and leaf N content were also significantly affected by the interactions of grazing and soil moisture.

**Figure 2:**
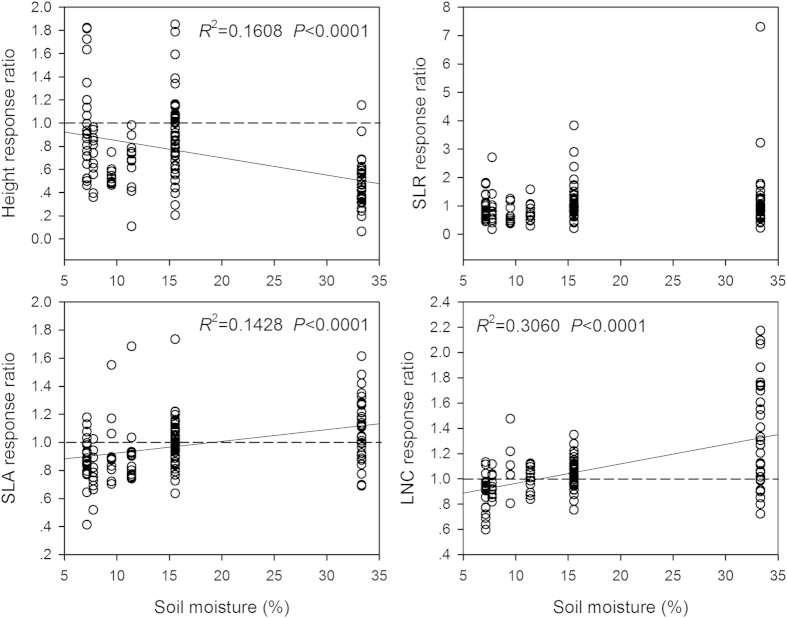
Relationships between the responses of plant functional traits to grazing and soil moisture. The response ratio of each trait was calculated as the ratio of mean values in the grazed to ungrazed communities (e.g. SLA _grazed_/SLA _ungrazed_). The reference line (dashed line) in each panel indicates no change in response ratio of trait. Abbreviations: SLR, stem-leaf biomass ratio; SLA, specific leaf area; LNC, leaf N content. Given that the five functional traits (i.e. plant height, individual biomass, SLR, SLA, and LNC) were correlated to a certain degree, we used Bonferoni correction in multiple statistical tests and the adjusted *P* value was 0.01 (Bonferroni corrected *P* = α/N = 0.05/5 = 0.01).

**Figure 3:**
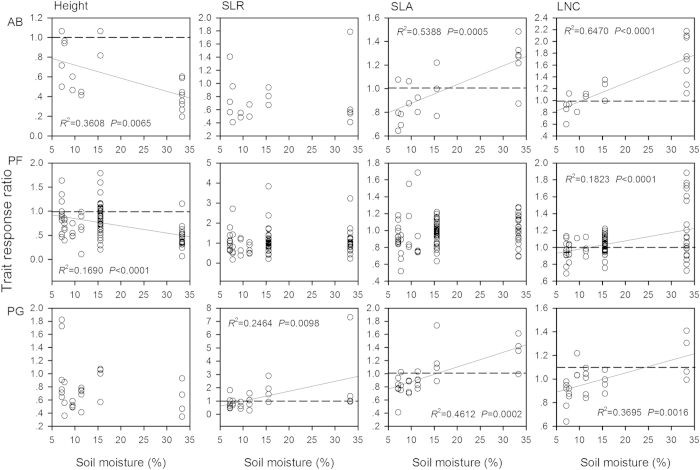
Relationships between the responses of plant functional traits of three life forms to grazing and soil moisture. The reference line (dashed line) in each panel indicates no change in response ratio of trait. Abbreviations: AB, annuals and biennials; PF, perennial forbs; PG, perennial grasses; SLR, stem-leaf biomass ratio; SLA, specific leaf area; LNC, leaf N content. The sampling sizes of three life forms are 20 species for annuals and biennials, 90 species for perennial forbs, and 26 species for perennial grasses. Given that the five functional traits (i.e. plant height, individual biomass, SLR, SLA, and LNC) were correlated to a certain degree, we used Bonferoni correction in multiple statistical tests and the adjusted *P* value was 0.01 (Bonferroni corrected *P* = α/N = 0.05/5 = 0.01).

**Figure 4:**
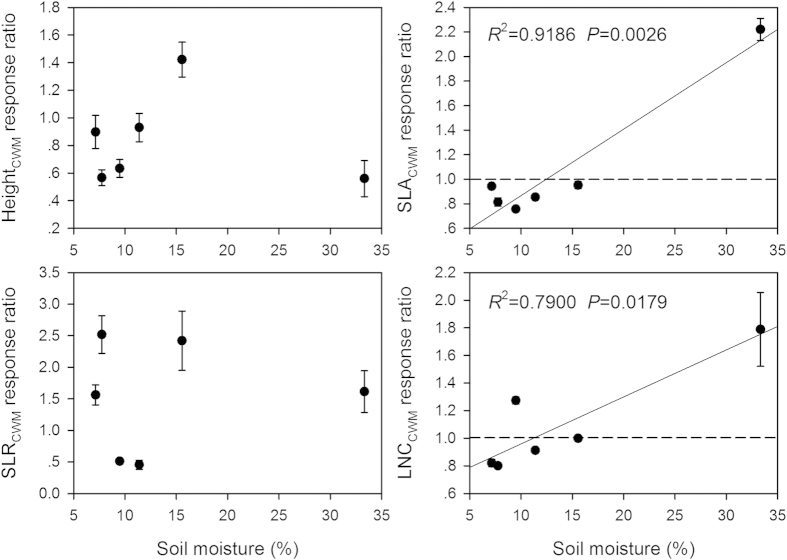
Relationships between the responses of community-weighted attributes to grazing and soil moisture. The reference line (dashed line) in each panel indicates no change in response ratio of community-weighted attribute. Abbreviations: SLR, stem-leaf ratio; SLA, specific leaf area; LNC, leaf N content; CWM, community-weighted mean.

The response ratio of plant height (PH_grazed_/PH_ungrazed_) to grazing decreased (*P* < 0.0001), while the response ratios of leaf N content (*P* < 0.0001) and SLA (*P* < 0.0001) increased along the soil moisture gradient (Fig. 2). The SLR and individual biomass response ratios didn’t show any trends along moisture gradient. Grazing diminished plant height but enhanced leaf N content and SLA for most species. For the three life forms, plant height response ratios of annuals and biennials and perennial forbs decreased, while SLR response ratio of perennial grasses increased along the moisture gradient (Fig. 3). The SLA response ratios of annuals and biennials and perennial grasses, and LNC response ratios of all three life forms increased along moisture gradient. But the individual biomass response ratios of all three life forms didn’t show any trends. At the community level, the response ratios of SLA_CWM_ and LNC_CWM_ to grazing increased greatly along the moisture gradient, while the response ratios of height_CWM_ and SLR_CWM_ didn’t show any trends (Fig. 4). Grazing increased the community-weighted SLA_CWM_ and LNC_CWM_ in wet sites but decreased them in dry sites.

### Variations in relative biomass of three life forms

**Figure 5:**
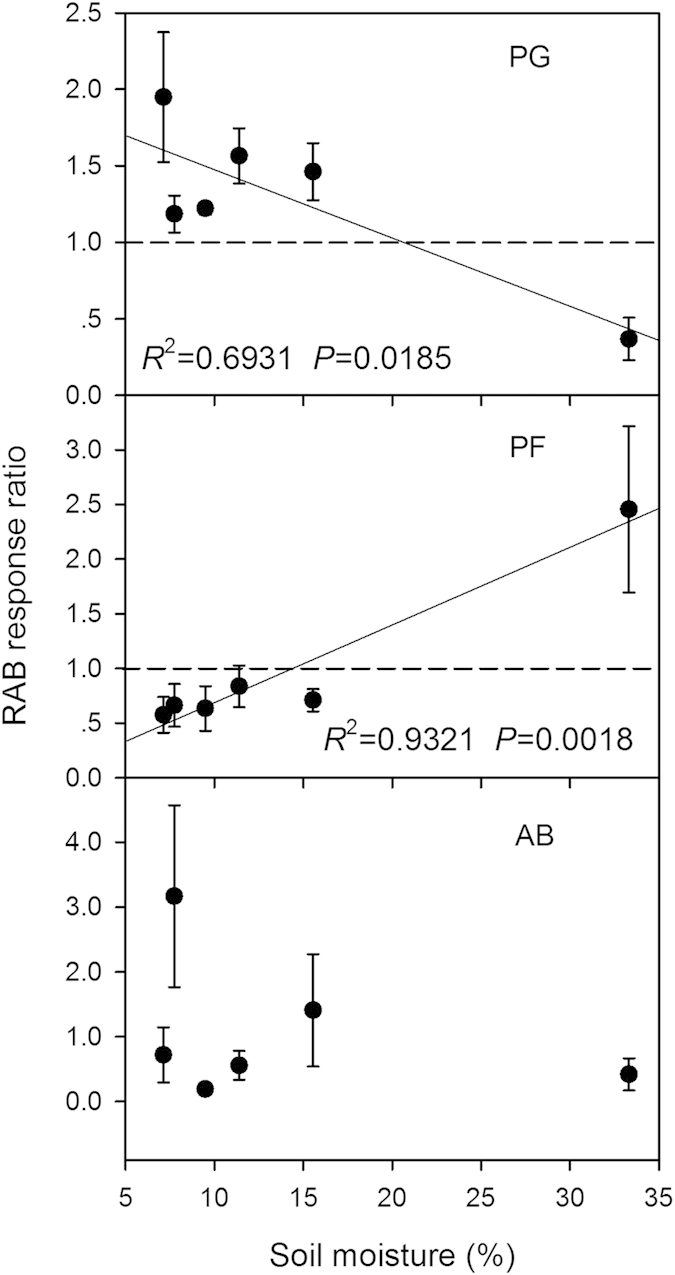
Relationships between the responses of relative aboveground biomass (RAB) to grazing and soil moisture. The reference line (dashed line) in each panel indicates no change in response ratio of relative biomass. Abbreviations: PG, perennial grasses; PF, perennial forbs; AB, annuals and biennials; RAB, relative aboveground biomass.

The relative biomass (RAB) response ratio of perennial grasses decreased significantly, while RAB response ratio of perennial forbs increased greatly along the moisture gradient (Fig. 5). Grazing decreased the relative biomass of perennial forbs in dry sites but increased it in wet sites. In contrast, grazing increased the relative biomass of perennial grasses in dry sites but decreased it in wet sites. The RAB response ratio of annuals and biennials varied little along the moisture gradient.

### Species turnover and intra- and inter-specific variability in SLA

**Figure 6:**
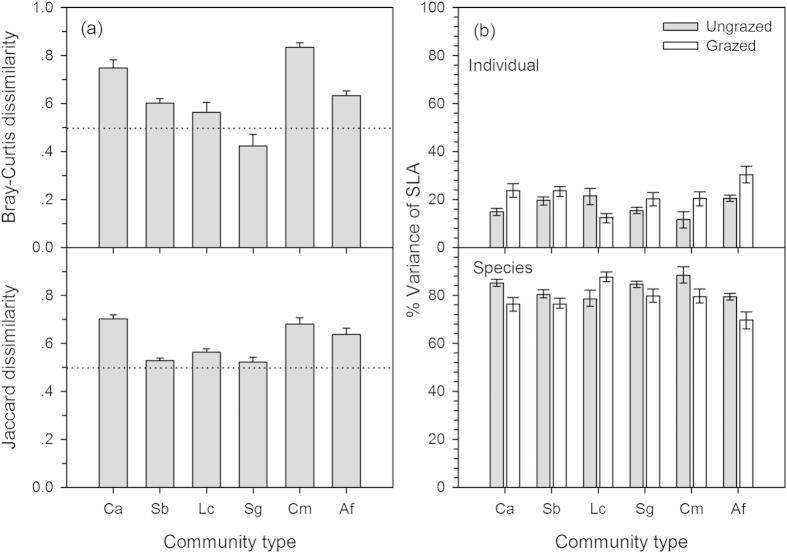
Variations of species dissimilarity (**a**) and effects of grazing on the variance partitioning of intraspecific variability (among individuals within species) and interspecific variability (among species) of specific leaf area (**SLA,b**) in six plant communities. In Fig. 6a, the reference line (dashed line) in each panel indicates the species dissimilarity index is 0.5. In Fig. 6b, error bars correspond to 95% confidence interval. Abbreviations of six communities: Ca, *Carex appendiculata*; Sb, *Stipa baicalensis*; Lc, *Leymus chinensis*; Sg, *S. grandis*; Cm, *Caragana microphylla*; Af, *Artemisia frigida*.

Two dissimilarity indices were used to identify species turnover due to grazing. Bray-Curtis dissimilarity between the ungrazed and grazed communities ranged from 0.42 to 0.83, with an average of 0.63, and Jaccard dissimilarity varied from 0.52 to 0.70 and averagely 0.61 (Fig. 6a). Both the dissimilarity indices were averagely higher than 0.6, indicating a remarkable species turnover had occurred in grazed communities.

The intraspecific variability (among individuals within species) explained 12–22% and interspecific variability (among species) explained 78–88% of the total variance in SLA across six ungrazed communities (Fig. 6b). For six grazed communities, the intra- and inter-specific variability separately explained 13–30% and 70–88% of the total variance in SLA. Based on 95% confidence intervals estimated by bootstrapping, grazing significantly increased the intraspecific variability of SLA by 20–77% in five of six communities.

### Effect of grazing on SLAs of dominant perennial grasses

**Figure 7:**
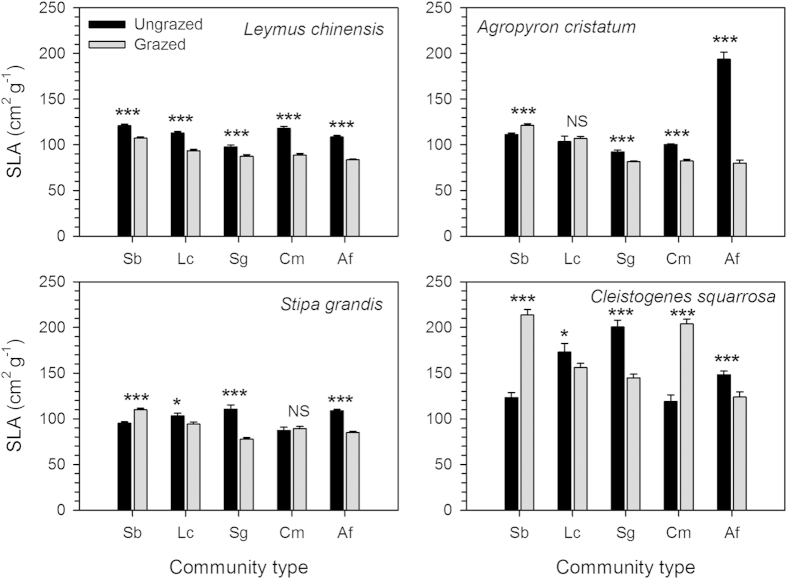
Effects of grazing on specific leaf area (SLA) of four dominant grasses in the meadow steppe and typical steppe communities. Abbreviations of five communities: Sb, *Stipa baicalensis*; Lc, *Leymus chinensis*; Sg, *S. grandis*; Cm, *Caragana microphylla*; Af, *Artemisia frigida*. Significant differences between the grazed and ungrazed communities are reported from ANOVA as NS, *P* > 0.1; *P < 0.05; ***P < 0.001. The error bars denote SE (*n* = 30).

The SLA of *Leymus chinensis* (a dominant perennial rhizomatous grass) was decreased by grazing in the meadow steppe and four typical steppe communities (Fig. 7). For the three dominant perennial bunchgrasses, i.e. *Stipa grandis, Agropyron cristatum* and *Cleistogenes squarrosa*, however, grazing increased their SLAs in relatively moist meadow steppe but decreased them in three of four dry typical steppe communities.

## Discussion

Our results demonstrated that two functional trait-based spectrums, i.e. individual biomass associated with plant size (PC1 axis) and leaf N content and leaf density related to leaf nutrient acquisition and shoot growth (PC3 axis), separated species from the grazed and ungrazed plant communities. These results suggest that plant species may have developed both tolerance strategies (e.g. increased leaf N content and leaf density) to improve shoot regrowth after defoliation^14^,^15^ and resistance strategies (through avoidance, e.g. decreased plant size) to reduce herbivore selectivity^9^,^10^,^11^. The variations in functional traits clearly indicate the fundamental trade-offs between productivity and persistence in plant functioning^37^,^38^, and further reflect the contrasting species-specific tolerance and defense strategies to grazing^39^,^40^,^41^,^42^. This study also suggests that the effects of grazing on plant functional traits are mediated by soil moisture, as indicated by the significant interactions of grazing and soil moisture on plant height, leaf N content and SLA. These results are consistent with previous studies^2^,^5^,^10^,^17^,^18^, which suggest that functional trait responses to grazing are largely dependent on resource availability.

In this study, we found that the effects of grazing on functional traits at the community level are dependent on plant functional group identity. This is supported by the evidence that the decrement in plant height was attributable to the negative responses of annuals and biennials and perennial forbs to grazing. The increments in leaf N content and SLA were due to the positive responses of all three life forms (except perennial forbs for SLA). The effects of grazing on community-weighted attributes are also dependent on plant functional group identity. The increments in SLA_CWM_ and LNC_CWM_ were mainly attributable to the increase in relative abundance of perennial forbs, and the positive trait responses of annuals and biennials and perennial grasses to grazing. These findings support our original hypothesis and further suggest that functional trait responses to grazing are largely mediated by both resource availability^2^,^5^,^10^,^17^,^18^ and functional group identity^29^,^30^. In this study, we also found grazing caused a shift in dominance from perennial forbs to perennial grasses in dry sites, as indicated by the increased relative abundance of perennial forbs but decreased relative abundance of perennial grasses with increasing soil moisture. This is because perennial forbs with low palatability (e.g. secondary metabolites with odor and toxins, spines and hairiness) showed more avoidance strategies to grazing^43^,^44^, which allow them to achieve rapid growth when other species were depressed by grazing, especially in moist and fertile habitats. In contrast, most perennial grasses in the Inner Mongolia grassland are xerophytes, which have long life-history, low growth potential (e.g. low leaf N and SLA), and great belowground competitive ability (e.g. high root: shoot ratio and root N content)^45^. Hence, these species have developed more conservative resource-use strategies and strong drought tolerance in the long-term process of evolutionary adaptation. In addition, perennial grasses are generally reproduced by germination of dormant buds and can develop new tillers rapidly^46^. Thus, these species generally show rapid regrowth capacity and more tolerance to grazing.

Our study suggests that species turnover and intensified intraspecific trait variability are the major drivers for the increased community-weighed SLA_CWM_ in response to grazing. This is supported by the results that Bray-Curtis dissimilarity and Jaccard dissimilarity in species composition (a measure of *β*-diversity) were averagely 0.61–0.63, indicating remarkable species turnover had occurred in grazed communities. In addition, the intraspecific trait variability explained 12–22% of the total variance in SLA, and grazing enhanced the intraspecific variability by 20–77%. This suggests that plant species exhibit high plastic adjustments in response to grazing, which may be related to phenotypic plasticity and genetic diversity^47^. Despite relatively lower magnitude of intraspecific trait variability compared to interspecific variatility (78–88%), it plays an important role in governing community-weighed SLA_CWM_ response to grazing. Our results imply that grazing promotes the intraspecific trait variability, which may benefit species coexistence and ecosystem stability^31^,^32^,^48^ and hence alleviate the negative impacts of grazing. Furthermore, the shifts in species composition due to grazing could be overestimated if we do not consider the intraspecific trait variability. These results are corroborated by previous studies, which suggest that intraspecific trait variability could mediate the functional responses of plant communities to environmental regimes, such as drought^32^, mowing and fertilization^33^. Our results provide the direct evidence that grazing modifies intraspecific trait variability and thereby community functional responses.

In this study, we demonstrated that the dominant perennial bunchgrasses, such as *Stipa grandis, Agropyron cristatum*, and *Cleistogenes squarrosa* exhibited mixed tolerance–resistance strategies to grazing and mixed resource acquisitive–conservative strategies to variation in water availability. This is evidenced by the results that grazing increased SLAs of these species in the moist meadow steppe, but decreased them in the dry typical steppe communities. It is widely documented that SLA is positively linked to potential growth rate^12^,^13^, resource acquisitive strategies^22^,^23^, and plant tolerance to grazing^9^,^42^. High leaf mass per area (LMA, an inverse of SLA), in contrast, is tightly associated with physical toughness^12^,^24^, resource conservative strategies^22^,^23^, and plant resistance to grazing^49^. Our results suggest that the dominant perennial bunchgrasses select resource acquisitive and grazing-tolerance strategies in wet and fertile habitats, but resource conservative and grazing-resistance strategies in dry and infertile habitats. In contrast, the dominant perennial rhizomatous grass, *Leymus chinensis*, didn’t show any mixed strategies of resource-use and herbivore-defense, as indicated by the decreased SLA values in all meadow steppe and typical steppe communities. These results were partially inconsistent with the prediction of our third hypothesis, we found among perennial grasses, only perennial bunchgrasses exhibited mixed strategies to grazing and resource variation. Our findings agree with a newly viewpoint that natural selection imposed by herbivores favors the evolution of mixed defensive strategies in plants, which have an intermediate level of tolerance and resistance^25^. Our results also showed that grazing increased the relative abundance of perennial bunchgrasses in dry sites, implying that the selective pressure imposed by long-term grazing and fluctuations in water availability may together promote the mixed strategies of plant anti-herbivore defense (tolerance–resistance) and resource utilization (acquisitive–conservative) in the Inner Mongolia grassland. Therefore, the results from this study provide new insights into the mechanisms of plant responses to grazing and variation in water availability in the arid and semiarid grasslands.

## Methods

### Study area

The study was conducted in the Xilin River Basin of the Inner Mongolia grassland (43°26′–44°29′N, 115°32′–117°12′E), which covers an area of 10 786 km^2^ with elevation ranging from 983 to 1469 meters above sea level^50^. The climate is semi-arid continental temperate steppe, with mean annual temperature of 0.4 °C and mean annual precipitation of 336.9 mm yr^−1^. Annual precipitation decreases gradually from 400 mm in the south-east to 250 mm in the north-west, and more than 80% of precipitation occurs in the growing season (May–August). Chestnut and dark chestnut soils are the zonal soil types in this region^50^. In this study, six pairs of parallel grazed and ungrazed plant communities were selected along a soil moisture gradient, including *Carex appendiculata* meadow, *Stipa baicalensis* meadow steppe, *Leymus chinensis* typical steppe, *S. grandis* typical steppe, *Caragana microphylla* typical steppe, and *Artemisia frigida* typical steppe^51^. The six plant communities are subjected to similar climatic conditions (i.e. temperature and precipitation), but they differ in soil moisture and other soil properties (e.g. soil organic carbon and nitrogen contents) (Supplementary Table S2). This is mainly caused by topography-controlled wind and water erosion and deposition processes^52^,^53^. The *C. appendiculata* meadow has the highest soil moisture and nutrients (e.g. soil organic matter and total nitrogen contents), followed by the *S. baicalensis* meadow steppe, and the four typical steppe communities have lower soil moisture and nutrients (Supplementary Table S2). The ungrazed sites of the six communities are the permanent field sites of the Inner Mongolia Grassland Ecosystem Research Station (IMGERS), Chinese Academy of Sciences, which have been fenced from grazing for about 20–30 years (Supplementary Table S2). In contrast, the grazed sites, located outside the fence of ungrazed sites, were managed as free grazing pasture. Sheep grazing has been the dominant form of land use practices in this area since 1950s. Seventy-eight to ninety-one percent of the total livestock numbers was composed of sheep, with numbers of cattle accounting for 5–9% and horses accounting for 2–13% of the total livestock numbers^54^.

### Vegetation and soil properties

The vegetation composition of the six plant communities were investigated from 28 July to 14 August, 2007, corresponding to annual peak aboveground biomass in this area^55^. At each site, 5–10 quadrats (1 × 1 m each) were located randomly within an area of 100 m ×100 m. Ten quadrats were used for meadow steppe and typical steppe, and 5 quadrats were for the more homogeneous meadow community. For the grazed sites, these quadrats were randomly located in the areas that were not subjected to grazing during the current growing season. Within each quadrat, all living biomass and current-year dead materials were harvested, separated to species, and oven dried at 70 °C for 24 h to constant mass and weighed. Litter biomass within each quadrat was also collected. The number of individuals and aboveground biomass of each species were measured to estimate species abundance and community standing biomass. Soil samples within each quadrat were collected by taking three 5-cm diameter soil cores from 0–20 cm depths, mixed *in situ* as one composite sample, and air-dried for soil nutrient analyses. Soil organic carbon was analyzed using K_2_Cr_2_O_7_-H_2_SO_4_ oxidation method, and total nitrogen was determined using the Kjeldahl acid-digestion method with an Alpkem autoanalyzer (Kjektec System 1026 Distilling Unit, Sweden). Subsamples of 0–20 cm soil layer were also collected, oven-dried at 105 °C for 48 h, and weighed to determine soil moisture.

### Plant functional traits

After vegetation survey, plant samples were collected at each site for functional trait measurements. A total number of 276 species were sampled across six paired plant communities, with 149 species in the ungrazed sites and 127 species in the grazed sites. There were 113 shared species in both the ungrazed and grazed sites. All species were classified into four functional groups primarily on the basis of life forms: perennial grasses, perennial forbs, annuals and biennials, and shrubs and semi-shrubs^55^. For each ungrazed or grazed site, 10–30 fully grown individuals of each species were randomly selected. Nine plant functional traits were determined by using standard methods^7^. These traits include plant height, individual biomass, stem biomass, leaf biomass, stem-leaf biomass ratio, total leaf area and leaf density per individual, specific leaf area (SLA), and mass-based leaf N content. The sampling replicates for each species were different among the 9 functional traits, with 30 individual replicates for SLA, 5 of 30 individuals for leaf N content, and 10 of 30 individuals for other 7 traits.

Plant height was measured by the distance from the basal stem to the natural crown of each individual^7^. After the height measurement, aboveground part of each individual was collected and taken back to the laboratory for stem and leaf separation. All leaves of an individual were picked and the number of leaves was recorded for leaf density estimation. Then 3–5 fully expanded mature leaves were selected to determine the projected leaf area with a portable leaf area meter (Li-3100C, Li-Cor, Lincoln, NE, USA), and the samples were finally used for leaf N content analysis. The stem and leaf samples were oven-dried at 70 °C for 24 h to constant masses and weighted. Hence, individual leaf area, dry mass per leaf, stem biomass, leaf biomass, plant individual biomass, total leaf area and leaf density per individual could be calculated, and specific leaf area and stem-leaf biomass ratio were separately calculated as the ratio of leaf area to dry mass, and ratio of stem biomass to leaf biomass^7^. Leaf samples were ground to homogeneity with a ball mill (MM 2000, Retsch GmbH & Co, Haan, Germany) for analyzing N content. For each grazed and ungrazed site, the community-weighted attributes for plant height (height_CWM_), stem-leaf ratio (SLR_CWM_), specific leaf area (SLA_CWM_), and leaf N content (LNC_CWM_) were calculated as trait means weighted by the relative biomass of each species within each quadrat^6^.

### Statistical analysis

Statistical analyses were performed with the software SAS version 9.2 (SAS Institute Inc., Cary, NC, USA), R version 2.15.0 (The R Foundation for Statistical Computing, Vienna, Austria), and Multi-variate Statistical Package (MVSP, Kovach Computing Services, Anglesey, UK). To detect general trends in functional traits of plant species across ungrazed and grazed communities, we first organized the dataset into a single nine traits (variables) × 276 species (cases) matrix, on which we carried out a principal component analysis (PCA) based on the correlation matrix of variables^23^,^56^. The effects of grazing on loading scores along PC1, PC2 and PC3 axes were examined using Independent-Samples T test. The effects of grazing and soil moisture on five key functional traits (i.e. plant height, individual biomass, stem-leaf ratio, SLA, and leaf N content) were tested with the generalized linear mixed model (GLMM), using species and community as random factors, and grazing, soil moisture and their interactions as fixed effects. The relationships between the responses of these five traits to grazing and soil moisture were analyzed for all species and life forms by using linear regression model. The response ratio of each trait was calculated as the ratio of mean value in the grazed to ungrazed communities (e.g. SLA_grazed_/SLA_ungrazed_). The shrubs and semi-shrubs were excluded from analyses due to small sampling size (<5 species). Using regression analysis, we also examined the relationships between the response ratios of community-weighted attributes (height_CWM_, SLR_CWM_, SLA_CWM_, LNC_CWM_) and soil moisture, and relationships between relative biomass response ratios of different life forms and soil moisture. The effects of grazing on SLA values of four dominant species, that is, one perennial rhizomatous grass (i.e. *Leymus chinensis*) and three perennial bunchgrasses (i.e. *Stipa grandis, Agropyron cristatum* and *Cleistogenes squarrosa*) were examined by Independent-Samples T test.

Given that SLA was measured with 30 individual replicates for each species, we used this trait to test whether species turnover and intraspecific variability may be responsible for the observed community functional responses (e.g. SLA_CWM_) to grazing. Two dissimilarity indices, i.e. Bray-Curtis dissimilarity and Jaccard dissimilarity in species composition (a measure of *β*-diversity) between ungrazed and grazed communities were used to evaluate species turnover due to grazing^32^,^57^. Bray-Curtis dissimilarity was calculated based on the relative biomass of each species between the paired ungrazed and grazed communities, and Jaccard dissimilarity was calculated based on presence-absence data^32^. In order to compare the contribution of intraspecific variability and interspecific variability on SLA variation, we used the nested linear model to decompose the total variance into two hierarchical components, including ‘among species’ (i.e. interspecific trait differences) and ‘among individuals within species’ (i.e. intraspecific variability), as described in Messier *et al.*’ study^58^. The 95% confidence intervals of variance components were estimated by bootstrapping (500 runs with data points resampled randomly from the full dataset with replacement).

## Additional Information

**How to cite this article**: Zheng, S. *et al.* Functional trait responses to grazing are mediated by soil moisture and plant functional group identity. *Sci. Rep.* **5**, 18163; doi: 10.1038/srep18163 (2015).

## Supplementary Material

Supplementary Information

## Figures and Tables

**Table 1:** Results of generalized linear mixed model analyses on the effects of grazing (G), soil moisture (M), and their interactions on five plant functional traits.

**Source**	**Functional traits**	***df***	**Mean Square**	***F***	***P-*****value**
Grazing	PH	1	174.81	83.55	0.0000
	PB	1	171.40	9.59	0.0023
	SLR	1	170.39	0.51	0.4769
	SLA	1	163.17	4.99	0.0269
	LNC	1	166.85	2.23	0.1377
Soil moisture	PH	5	209.66	0.46	0.8023
	PB	5	210.92	3.12	0.0097
	SLR	5	207.49	6.09	0.0000
	SLA	5	200.42	28.10	0.0000
	LNC	5	197.99	9.09	0.0000
G × M	PH	5	174.81	11.99	0.0000
	PB	5	171.40	1.54	0.1793
	SLR	5	170.51	0.63	0.6773
	SLA	5	163.69	3.08	0.0111
	LNC	5	166.48	12.85	0.0000

Note: PH, plant height; PB, plant individual biomass; SLR, stem-leaf biomass ratio; SLA, specific leaf area; LNC, leaf nitrogen content.
