# Supplementary material for: Functional trait responses to grazing are mediated by soil moisture and plant functional group identity
Source: Sci Rep. 2015 Dec 11;5:18163. doi: 10.1038/srep18163 (PMC4676060; doi:10.1038/srep18163)
Supplement: Supplementary Information [file srep18163-s1.pdf]

Functional trait responses to grazing are mediated by soil moisture and plant functional group identity

Shuxia Zheng, Wenhuai Li, Zhichun Lan, Haiyan Ren, and Kaibo Wang

Supplementary Information

Table S1. Principal components analysis (PCA) of species-trait correlation matrix. All variables were log10-transformed before analysis. Eigenvectors >|0.40| are highlighted in bold. Percents reflect the percent of total variance (e.g. the sum of the diagonal elements in the correlation matrix) accounted for by each principal component.

	**PC1**	**PC2**	**PC3**
Eigenvalues	3.91	1.71	1.22
% of Variance	43.43	19.03	13.50
Cumulative % variance	43.43	62.46	75.96
Eigenvectors			
Plant height (PH)	0.211	**0.420**	**-0.419**
Plant individual biomass (PB)	**0.497**	0.004	-0.018
Stem-leaf biomass ratio (SLR)	0.067	**0.661**	-0.024
Stem biomass (SB)	**0.466**	0.198	0.005
Leaf biomass (LB)	**0.472**	-0.217	-0.009
Total leaf area (TLA)	**0.432**	-0.084	0.044
Leaf density (LD)	0.269	-0.192	**0.494**
Specific leaf area (SLA)	-0.052	**0.424**	0.199
Leaf N content (LNC)	-0.056	0.277	**0.733**

Table S2. Locations, soil properties and land use type of six plant communities in the Xilin River Basin of Inner Mongolia, China.

No.	Community type	Vegetation type	Location	Altitude (m)	Soil moisture (%)	Soil organic matter (g kg^{-1})	Soil total nitrogen (g kg^{-1})	Land use type in ungrazed site
1	*Carex appendiculata*	Meadow	43°38′N 116°41′E	1150	33.32	121.20	5.25	Fenced since 1989
2	*Stipa baicalensis*	Meadow steppe	43°27′N 116°47′E	1380	15.55	32.41	1.87	Fenced since 1979
3	*Leymus chinensis*	Typical steppe	43°33′N 116°41′E	1250	11.40	28.45	1.58	Fenced since 1979
4	*S. grandis*	Typical steppe	43°33′N 116°33′E	1180	9.49	28.45	1.71	Fenced since 1979
5	*Caragana microphylla*	Typical steppe	43°36′N 116°44′E	1190	7.75	21.21	1.19	Fenced since 1983
6	*Artemisia frigida*	Typical steppe	43°38′N 116°41′E	1200	7.14	13.10	0.71	Fenced since 1989
